# Food addiction in a large non-clinical sample of Canadians

**DOI:** 10.1192/j.eurpsy.2021.2181

**Published:** 2021-08-13

**Authors:** A. Samokhvalov, C. Murphy, I. Balodis, J. Mackillop

**Affiliations:** 1 Department Of Psychiatry And Behavioral Sciences, McMaster University, Hamilton, Canada; 2 Centre For Alcohol And Addiction Studies, Brown University School of Public Health, Providence, United States of America

**Keywords:** quality of life, Impulsivity, food addiction, obesity

## Abstract

**Introduction:**

The concept of food addiction emerged recently due to the similarities between food overconsumption patterns and addictive drugs. This concept is not yet included into ICD or DSM as it still needs to be further investigated. Relationship between obesity and food consumption as well as the psychological indicators of food addiction are of particular interest.

**Objectives:**

To examine the prevalence of food addiction and its relationship to obesity, quality of life and multiple indicators of impulsivity.

**Methods:**

Cross-sectional in-person assessment of 1432 community adults (age 38.93+/-13.7; 58% female). Measurements: Yale Food Addiction Scale 2.0, anthropometrics, body composition, World Health Organization Quality of Life scale, and impulsivity measures including impulsive personality traits, delay discounting, and behavioral inhibition.

**Results:**

The prevalence of food addiction was 9.3% and substantially below that of obesity (32.7%). Food addiction was more prevalent among obese individuals and also was associated with higher BMI among non-obese participants. It was associated with significantly lower quality of life in all domains, and significantly higher impulsive personality traits, particularly negative and positive urgency.

**Conclusions:**

In this general community sample, food addiction was present in slightly fewer than 1 in 10 individuals, approximately one-third the prevalence of obesity, but notably the food addiction has been mostly represented within the subsample of obese individuals. Food addiction was robustly associated with substantively lower quality of life and elevations in impulsivity, particularly in deficits in emotional regulation. These data suggest food addiction may be thought of as a subtype of obesity and, in non-obese individuals, possibly a prodrome.

**Disclosure:**

No significant relationships.

